# Heat Shock Proteins Can Be Surrogate Autoantigens for Induction of Antigen Specific Therapeutic Tolerance in Rheumatoid Arthritis

**DOI:** 10.3389/fimmu.2019.00279

**Published:** 2019-02-22

**Authors:** Willem van Eden, Manon A. A. Jansen, Irene S. Ludwig, Paul Leufkens, Marlies C. van der Goes, Jacob M. van Laar, Femke Broere

**Affiliations:** ^1^Infectious Diseases and Immunology, Utrecht University, Utrecht, Netherlands; ^2^Faculty of Veterinary Medicine, Department of Infection and Immunity, Utrecht University, Utrecht, Netherlands; ^3^University Medical Center Utrecht, Utrecht, Netherlands

**Keywords:** stress protein, heat shock protein, rheumatoid arthritis, tolDC, autoimmunity

## Abstract

Technologies that enable induction of therapeutic tolerance may revolutionize the treatment of autoimmune diseases by their supposed potential to induce drug-free and lasting disease remission. In combination with diagnostic tests that screen for individuals at risk, these approaches may offer chances to halt disease before serious damage in the tissues can occur. In fact, for healthy individuals at risk, this could lead to a preventive form of vaccination. For therapeutic tolerance to re-instate natural self-tolerance it seems essential to induce tolerance for the critical autoantigens involved in disease. However, for most autoimmune diseases such antigens are poorly defined. This is the case for both disease inciting autoantigens and antigens that become involved through epitope spreading. A possible source of surrogate auto-antigens expressed in tissues during inflammation are heat shock proteins (HSP) or stress proteins. In this mini-review we discuss unique characteristics of HSP which provide them with the capacity to inhibit inflammatory processes. Various studies have shown that epitopes of HSP60 and HSP70 molecules can function as vaccines to downregulate a variety of autoimmune inflammatory diseases. Currently, several research groups are developing cell therapies with the intention to reach therapeutic tolerance. In this review, in which we are proposing to *ex vivo* load tolerant dendritic cells with a Treg inducing HSP70 derived peptide called B29, we are discussing the chances to develop this as an autologous tolDC therapeutic tolerance therapy for rheumatoid arthritis.

## Introduction

Although originally discovered by their enhanced expression following the rise of temperature, the enhanced expression of HSP is now known to depend on a multitude of factors which include the mediators associated with inflammation. Research directed toward the role of HSPs in inflammatory diseases has put emphasis on the involvement of the HSP60, HSP70, and HSP90 molecules. In the new nomenclature these molecules are known as HSPD, HSPA, and HSPC (1). These families of larger HSPs tend to display strong evolutionary conservation from bacteria to humans (2). Recognizing the role of immune encounters with microbiota, such as in the tolererizing gut mucosal tissues, suggests that due to this evolutionary conservation T cells adopt a tolerizing phenotype upon their encounter with these abundant and omni-present molecules. The larger family of HSP molecules is the HSP70 or HSPA family. Some HSP70 proteins are constitutively expressed, while some others are highly stress inducible.

## Expression of Heat Shock Proteins in Stressed Cells and Inflamed Tissues

Peptide elution profiles obtained from MHC class II molecules have revealed the frequent presence of HSP70 derived peptides in MHC-II. This was in particular the case for MHC-II of stressed cells, possibly resulting from the known role of HSP70 proteins in the process of chaperone mediated autophagy. Paludan et al. (3) have analyzed the proteomes of both MHC-I and MHC-II molecules during virus infection, both mouse and human. In this study the three most frequent nuclear/cytosolic natural ligand sources that were defined for MHC-II were HSC70, HSP70, and GAPDH. Another study by Dengjel et al. (4) analyzed the proteome of an HLA-DR4 molecule obtained from human B cells stressed by nutrient deprivation. Also in this study the dominant presence of HSP70 fragments in this proteome was said to result from autophagy. Interestingly enough, one of the HSP70 peptides that was eluted appeared to be identical with a peptide, called mB29b, representing an epitope recognized by regulatory T cells earlier defined by us in our experimental arthritis model (5). A report dealing with the enhanced expression of HSP in tissues during inflammation can be found in Schett et al. (6). In this study expression of HSP70 was shown for the synovial tissue of RA joints. In addition the effect of fever and TNFα driving the cellular expression of HSP70 and its transcription factors was shown.

## Tolerance Driven by Microbial—Mammalian HSP T Cell Cross-Recognition

The first evidence for tolerizing effects of HSP was obtained in the model of heat-killed mycobacteria induced arthritis in Lewis rats, the model of adjuvant arthritis (7). We cloned HSP60 from mycobacteria and showed that this antigen was the main T cell target in the model. However, HSP60 immunization did not produce any arthritis and such immunized animals were shown to become resistant to induction of adjuvant arthritis. Interestingly, HSP60 immunization also was shown to protect in other models, such as arthritis induced with streptococcal cell walls (8) and arthritis induced with an oily compound called pristine (9). Besides arthritis, also models of diabetes and atherosclerosis showed the protective effects of mycobacterial HSP60 [reviewed in van Eden et al. (10)]. By analyzing T cell epitopes of mycobacterial HSP60 in Lewis rats we detected the 256–265 sequence being very conserved. T cells specific for 256–265 cross-responded to the mammalian HSP60 molecule and were found to protect against disease upon adoptive transfer in the adjuvant arthritis model (11). T cells raised against non-conserved epitopes did not protect. Similar results were obtained more recently for HSP70 in a model of proteoglycan induced arthritis in mice. In this model, the B29 peptides, of which mB29b was already mentioned here above to be present in the MHC-II elution profile of stressed cells, were found capable of inducing protective T cells based on microbial-mammalian cross-recognition (5).

Before introducing the possible mechanisms leading to the tolerance promoting activities of HSP peptides we describe in more detail our B29 peptide. This peptide of mycobacterial origin is 15 amino- acids long with the following sequence: VLRIVNEPTAAALAY. Its mammalian homolog mB29b: VLRIINEPTAAAIAY. Both peptides were high to moderate MHC binders, when tested in competitive binding assays, for most human HLA class II molecules, such as HLA-DR1, DR3, DR4, DR11, DQ2, and DQ8 (12). Their functionality and protective effects in the context of human HLA molecules were shown in a model of proteoglycan induced arthritis (PGIA) carried out in a HLA-DQ8 transgenic mouse (12). Also T cell responses in humans with specificity for these peptides, including cross-reactivities between B29 and its mammalian homologs, were documented (12).

How the microbiota impacts the immune system has been analyzed in great detail over recent years. For instance, mucosal dendritic cells (DC) are now known to regularly sample the microbiota and to present their antigens to T cells (13). It may be inferred that the bacterial stress response that evolves following ingestion leads to upregulation of microbial HSP and that therefore microbial HSP peptides are frequently seen by the mucosally residing Tregs. Along similar lines it can be understood that the more conserved peptide sequences will dominate, given their more frequent and repetitive presence in the antigenic make-up of our diverse microbiota. In this manner it is possible that the basis for induction of Treg responses by conserved HSP peptides is imprinted into the immune system at the mucosa or other sites of the body where contact with microbiota occurs. Therefore, it is possible that a combination of stress inducibility and the evolutionary conservation has provided HSP with the capacity to control inflammation.

## HSP Dampen Immune Responses

Since the molecular cloning of HSP60 from mycobacteria, many studies were done with recombinant, in *E. coli* produced, HSP proteins. Insufficient attempts to clear these proteins from contaminating LPS has resulted in false positive observations and reports of immune activation. However, several subsequent studies have shown that pure HSP preparations were lacking immune stimulating activities (14). Nonetheless, in too many cases HSP are listed as example molecules with damage associated molecular patterns or DAMPs. Apart from the fact that clean molecules were shown to lack DAMP qualities, many arguments exist that further disqualify HSP for being DAMPs (15). By their nature DAMPs are exclusively intracellular and are supposedly only released by cells upon damage. HSP, however, are known to be present in body fluids, such as for example serum. Furthermore, it was shown in various studies that HSP can have immuno-modulatory effects on DC. In the mouse PGIA model, HSP70 treated DCs loaded with proteoglycan, were found, upon *in vivo* transfer, to suppress disease. In addition, HSP70 treated DC loaded with OVA, were found to induce production of IL-10 in OVA specific T cells (16). These findings were made with both mycobacterial and murine HSP70. In a different set of experiments carried out by others, mycobacterial HSP70 was shown to impair the maturation of bone marrow derived DC, to induce IL-10 production and to inhibit T cell proliferation (17). These findings, together with the reported disease inhibitory activities of HSP molecules, are pleading against HSP being DAMPs, but indicative of their immune DAMPing capacities instead.

## Rheumatoid Arthritis as a Model Autoimmune Disease

Despite recent advances in the treatment of RA using a range of biological therapies (for example anti-TNF, CTLA-4, and anti-B-cell therapies), very few patients achieve long-term clinical remission, even when therapies are started early. A major challenge for research and drug development is now to find ways to change the outcome, with the aim of reaching sustained remission or cure in a large majority of patients. Patients that benefit from biological therapies are treated with weekly to monthly/half-yearly injections and continued use of oral methotrexate, with unpleasant side-effects, and sometimes serious and life-threatening adverse effects due to suppression of the immune system. The ultimate therapeutic ambition for rheumatologists is to provide drug-free remission for all patients. Theoretically this could be achieved using a short course or infrequent (vaccine-like) treatment to restore normal immunity and prevent further synovial damage to maintain joint function. Such a therapy would not only benefit patients with established RA, it could also be used before the onset of RA to halt the disease process at the early immune initiation phase of the disease, before any joint damage has occurred. A summary of clinical trials developed for HSP based interventions in RA is given in the Table 1.

**Table 1:** HSP based clinical trials in inflammatory arthritis.

**HSP product**	**Nr of patients**	**Administration**	**Effect**	**References**
HSP40 (DnaJ) peptide: DnaJP1	RA *N* = 15	Oral 6 months	Increased IL-4 and IL-10. Decreased IL-2, IFN-γ, TNF and T cell proliferation	(18)
HSP40 (DnaJ) peptide: DnaJP1	RA *N* = 160	Oral 6 months	Clinical response, ACR20. Less T cells producing TNF	(19)
HSP10 (Chaperonin10)	RA *N* = 23	Intravenous 12 weeks (twice in a week)	Clinical improvement of disease activity	(20)
HSP70 (BiP)	RA *N* = 24	Intravenous (single administration)	Some patients with clinical and biological improvements	(21)

The HSP10 study was lacking a placebo group, which impaired full interpretation of results. The most recent study with HSP70 showed significantly prolonged remissions at the highest protein concentrations administered (5 and 15 mg). This in itself has suggested the induction of Treg, supporting the original hypothesis regarding the therapeutic action of HSP.

Improved understanding of the role of regulatory T cells and dendritic cells in suppressing the immune response may also lead to novel therapeutics to induce immune tolerance. Immune tolerance could be achieved using a combination of existing therapies, novel drugs and cell-based therapies and peptide immunotherapy to re-regulate and suppress the pathogenic immune response in RA. RA is an ideal disease setting for the study of tolerance inducing therapies. This is due to recent insights into the immunology of RA, coupled with advances in autoantibody identification and T and B-cell monitoring.

## A Cell Therapy Approach in Combination With HSP for Rheumatoid Arthritis

**Figure 1:**
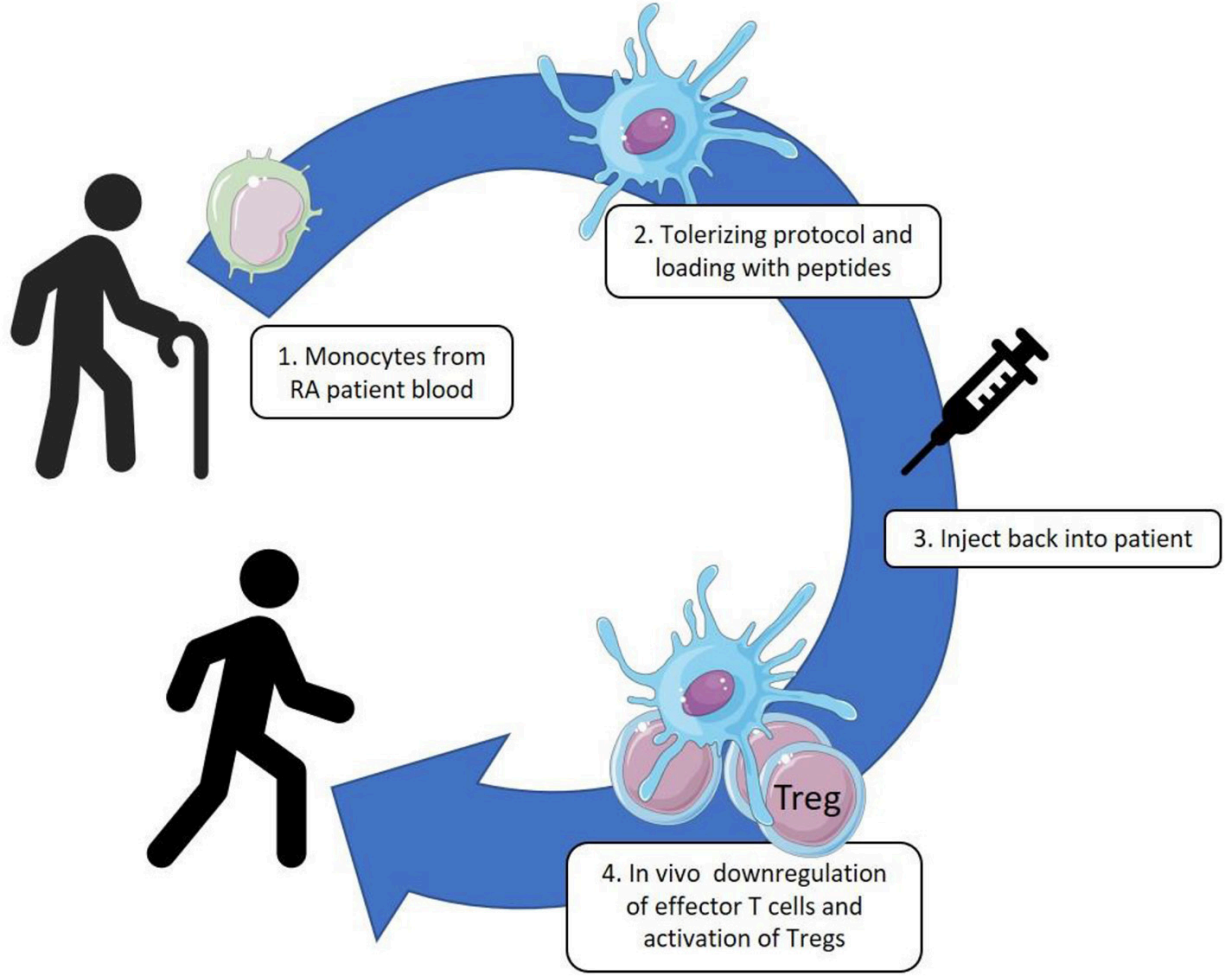
Monocytes are collected from patient blood by leukapheresis and cultured with GM-CSF. These cultured DC-like cells are treated with VitD3-Dexamethasone, matured by monophosphoryl lipid A (MPLA) and loaded with the HSP70-B29 peptide. The tolerized and antigen loaded DC are injected back into the patients by intracutaneous or intranodal delivery.

To develop a lasting cure through tolerance therapy for RA is an attractive, but certainly great challenge for immunologists. Since the discovery of dendritic cells as central elements in the initiation of cellular immune responses, the idea has surfaced that DCs are critical in the organization of tolerance and that resting tissue DCs may well have a role in presenting autoantigens in default manner for inspection by Tregs. Be that as it may, antigen presentation by DCs in a suboptimal manner may lead to induced Tregs (iTreg) (22), and as a consequence such iTreg do further impose tolerogenicity in other DCs, amongst others through their production of immunoregulatory cytokines, such as IL-10 and TGF-β. In this manner a self-sustaining cycle of tolerance induction may become operationally active. At the same time, through linked recognition, these cytokines may act on other naïve T cells and recruit them into the pool of iTregs, a process known as induction of infectious tolerance (23, 24). Through such mechanisms a lasting and spreading tolerance for a multitude of self-antigens may be created. In theory this situation may be imposed on an immune system in an inflammatory state, by instilling artificially tolerized DC into the system. The rheumatology group of Newcastle University (UK) has pioneered such approach in a first clinical trial (25). Monocyte derived DCs, taken from patients with RA, were cultured in the presence of VitD3 and dexamethasone, loaded with synovial fluid collected from patients and injected back into their inflamed joints. In this autologous cell therapy, tolerization was sought for the mixture of substances in the synovial fluids taken from inflamed joints which supposedly included critical RA auto-antigens. With this trial the logistics for such an approach were developed and the procedure was proven safe. However, with a complex mixture of antigens such as in synovial fluid, monitoring the immunological effect was complicated. Moreover, the necessity of including the patients with more advanced disease, with severely inflamed joints, for obtaining synovial fluids, was not advantageous. In collaboration with the Newcastle group we are now planning a similar trial with autologous tolDC, but now with HSP70 peptide B29 loaded tolDC (Figure 1). This Phase I-II, unblinded, longitudinal study will include 22 RA patients who will be treated with either a low dose (5 × 10.6) or a high dose (15 × 10.6) of cells intracutaneously or intranodal. The patients will be in remission or with low disease activity under conventional therapy. Exact monitoring of the effect of this tolerance therapy will be possible by tracing and characterizing B29 specific T cells following our B29 tolDC intervention. In other words, besides monitoring of adverse events as the primary outcome, we will monitor peptide specific T cell responses.

## Cell Therapy and Beyond

For the clinical use of our novel proposed therapy, the specificity of the induced effects with antigen loaded tolDC, is more appealing and likely more safe with less general immuno-suppression than we see with the currently available therapies. And moreover, already for the purpose of immune monitoring the antigen specificity of the intervention will be an essential and enabling aspect. An obvious possible limitation of the use of tolDC in chronic inflammatory disorders is that tolDC may not be able to utilize their tolerizing activities in the context of an ongoing inflammation. However, this potential limitation may possibly become negated by a synergistic effect of the intrinsic capacity of HSP to induce Tregs and of tolDC to convey immunological tolerance. As already mentioned by others, after biologics, the use of human cells as versatile therapeutic engines can be a new revolution in pharmaceutical practices (26). And especially for tolerance therapies the use of Tregs and tolDC may turn out to be effective approaches. Although costly, the prospect of lasting cure may well-compensate for high costs of tolerance therapies in chronic and severely debilitating disorders. For practical purposes however, the delivery of antigens directly *in vivo*, and directed to the relevant cells *in vivo* may be an attractive next step. To reach tolDC targeting *in vivo* it is for instance possible to exploit a novel tolerance-inducing technology that utilizes several components. Examples are Poly Lactic-co-Glycolic Acid (PLGA)-Polyethyleenglycol (PEG)-PLGA hydrogels for sustained presentation of peptides, liposomal vitamin D3 for enhanced tolerization of targeted peripheral APC, or PLGA particles loaded with TNFα-siRNA. In earlier studies we have seen that nasal administration of B29 in PLGA particles was more effective in the prophylactic suppression of arthritis in the PGIA model than peptide alone (27). In addition, PLGA particles were found to induce a tolDC-like phenotype by enhancing retinaldehyde dehydrogenase (RALDH) in DC and to upregulate Foxp3 in T-cells (28). Incorporating vitamin D3 liposomes in such a slow release matrix (hydrogels) can enhance *in vivo* induction of tolDC comparable to current *in vitro* culture protocols to induce tolDC for cell therapy (29). Treatment of epidermal Langerhans cells with the active form of vitamin D3 generates functional Foxp3^+^ Tregs through a mechanism dependent on keratinocyte-derived TGF-β. In contrast, treatment of dermal DCs with 1, 25-(OH)_2_vitamin D3 generates functional IL-10^+^FoxP3^−^ T_R_1 cells in an IL-10-dependent fashion (30).

In combination with tolDC interventions it may be needed to interfere with the production of pro-inflammatory cytokines such as TNF. Short interfering RNAs (siRNA) hold a promising therapeutic potential against a variety of disease conditions. However, actual potency seems limited by its physicochemical properties i.e., high hydrophilicity and poly-anionic phosphate backbone leading to negligible cellular permeation and subsequent siRNA delivery. Recently, lipid-polymer hybrid nanoparticles (LPNs) have been shown to act as efficient carriers for intracellular delivery of siRNA both *in vitro* and *in vivo* (31).

## Conclusion

Antigen specific tolerance therapies for autoimmune diseases are currently under development. In many cases however, the critical autoantigens are still escaping proper identification. Heat shock proteins are attractive molecules as surrogate auto-antigens and may have several advantages in comparison with regular autoantigens. Their conserved nature and their abundant presence in the MHC II peptidome of stressed cells in particular, seems to provide them with unique characteristics as targets for regulatory T cells. It is hoped that first clinical trials with HSP loaded tolDC, such as are now being developed for rheumatoid arthritis, will pave the way for further tolerance therapies, leading to medication free lasting remission of disease.

## Author Contributions

All authors listed have made a substantial, direct and intellectual contribution to the work, and approved it for publication.

### Conflict of Interest Statement

The authors declare that the research was conducted in the absence of any commercial or financial relationships that could be construed as a potential conflict of interest.
